# Integrated transcriptome and metabolome analysis provides insights into blue light response of *Flammulina filiformis*

**DOI:** 10.1186/s13568-024-01680-w

**Published:** 2024-02-13

**Authors:** Huan Wang, Shuting Zhao, Zhiyang Han, Zexin Qi, Lei Han, Yu Li

**Affiliations:** 1 Department of Agronomy, Jilin Agricultural University, Changchun, 130118 China; 2 Institute of Crop Science, Chinese Academy of Agricultural Sciences, Beijing, 100081 China; 3 Key Laboratory of Molecular Epigenetics of Ministry of Education, Northeast Normal University, Changchun, 130024 China; 4 Department of Plant Protection, Jilin Agricultural University, Changchun, 130118 China

**Keywords:** *Flammulina filiformis*, Morphogenesis, Blue light, Red light

## Abstract

Blue light promotes primordium differentiation and fruiting body formation of mushroom. However, the blue light response mechanism of mushroom remains unclear. In this study, mycelium of *Flammulina filiformis* was exposed to blue light, red light and dark conditions, and then the comparative metabolome and transcriptome analysis was applied to explore metabolic regulation mechanism of *F. filiformis* under blue light and red light conditions. The yield of the fruiting body of *F. filiformis* under blue light condition was much higher than that under dark and red light conditions. Metabolome analysis showed that blue light treatment reduced the concentrations of many low molecular weight carbohydrates in the pilei, but it promoted the accumulation of some low molecular weight carbohydrates in the stipes. Blue light also decreased the accumulation of organic acids in the stipes. Blue light treatment reduced the levels of tyrosine and tryptophan in the stipes, but it largely promoted the accumulation of lysine in this organ. In the stipes of *F. filiformis*, blue light shifted metabolite flow to synthesis of lysine and carbohydrates through inhibiting the accumulation of aromatic amino acids and organic acids, thereby enhancing its nutritional and medicinal values. The transcriptome analysis displayed that blue light enhanced accumulation of lysine in fruiting body of *F. filiformis* through downregulation of lysine methyltransferase gene and L-lysine 6-monooxygenase gene. Additionally, in the stipes, blue light upregulated many hydrolase genes to improve the ability of the stipe to biodegrade the medium and elevated the growth rate of the fruiting body.

**Supplementary Information:**

The online version contains supplementary material available at 10.1186/s13568-024-01680-w.

## Introduction

The yield and quality of mushroom are important factors for the development of edible mushroom industry. How environmental factors affect the yield and quality of mushroom is current research hot. Temperature, nutrient composition of medium, and light conditions are the key environmental factors for primordium differentiation and fruiting body induction of mushroom. With the exploitation of wild mushroom resources, more and more wild mushrooms are domesticated and cultivated. Most of these domesticated mushrooms, such as shiitake mushroom, enoki mushroom, morchella, cordyceps, black fungus and *Ganoderma lucidum*, need low temperature and lights to induce formation of primordium. It has been reported that lights not only have effects on mycelium and fruiting body formation, but also produce effects on quality and yield of mushrooms (Sakamoto 2018; Du et al. 2020; Ye et al. 2022), which has become research hot. Photoreceptors and light signaling mechanisms have been largely explored in fungi (Corrochano 2019; Yu and Fischer 2019; Bayram and Bayram 2023), but most mechanistic knowledge comes from a few filamentous fungi such as *Aspergillus nidulans* and *Neurospora crassa* and light signaling mechanism is poorly understood in mushroom (Bayram and Bayram 2023). Some studies have demonstrated that lights play a crucial role in the morphogenesis of mushroom fruiting bodies (Fuller et al. 2015; Sakamoto 2018). The induction of fruiting body by lights with different wavelength has been explored (Kitamoto and Gruen 1976; Durand and Furuya 1985), which indicated that the effective induction light was mainly ultraviolet light (280 nm) and blue light (520 nm). Light treatments also can improve the quality of preharvest and postharvest edible mushrooms (Fernandes et al. 2013; Feng et al. 2023a, b).

Previous studies have found that blue light has a strong effect on metabolism of mushroom fruiting bodies (Kojima et al. 2015). Recently, some studies explored regulation mechanism of gene expression of blue light response in mushroom (Sakamoto et al. 2018; Xie et al. 2018; Wang et al. 2020; Kim et al. 2021). Wang et al. (2020) found that blue light can promote the growth of fruiting bodies in oyster mushrooms by improving energy metabolic processes such as glycolysis and pentose phosphate pathway. Xie et al. reported that blue light regulates expression of CAZymes gene during primordium differentiation (Xie et al. 2018). Hu et al. (2018) found that white light and blue light treatments can enhance yield of the fruiting body, and red light and yellow light inhibited the growth of the fruiting body (Hu et al. 2018). Although the previous studies have explored the blue light response of mushrooms in different aspects, the metabolic regulation and molecular mechanism of the blue light response of mushrooms are still not fully understood. In addition, different species of mushrooms may display various blue light response mechanisms (Sakamoto 2018). Therefore, blue light response should be investigated in each case of mushroom.

Enoki mushroom (*Flammulina filiformis*) not only has high nutritional value, but also has high medicinal value. Fruiting body of *F. filiformis* not only displays high amino acid content but also contains a variety of amino acids. As *F. filiformis* contains high concentrations of lysine, arginine and methionine that can promote intellectual development, it is considered as “intellectual mushroom”. *F. filiformis* also contains high concentrations of antioxidant components, such as vitamin C, B vitamins and polyphenols, and enoki mushroom polysaccharide has also been demonstrated to have antiviral, anti-tumor and other medicinal functions. The above qualities largely enhance the market potential of enoki mushroom industry. In this paper, metabolome and transcriptome analyses were used to explore the physiological and gene expression regulation mechanisms underlying blue light response of fruiting body in enoki mushroom. Our results will provide technical supports for the development of edible mushroom industry.

## Methods

### Fruiting body cultivation and light treatments

*Flammulina filiformis* strain Chuanjin-4 was selected as test organism because it is a stable commercial strain available in Jilin Province, China. The *F. filiformis* strain was purchased from Luofeng Fungal Company (Chengdu, China). The inoculation was conducted according to the method of Wang et al. (2020). The prepared liquid spawn was inoculated into sterilized pots (240 mL, 90 mm height, and 65 mm diameter) containing the growth medium (65% moisture content) that consisted of wood chips (73%), wheat bran (25%), sucrose (1%), and CaCO_3_ (1%). The pots were placed in a mushroom incubation chamber (Hipoint Corporation, Taiwan) at 24 °C and dark condition for 30 days. After 30 days of cultivation, *F. filiformis* primordium emerged. The pots with newly emerged primordium were exposed to blue light, red light, and dark treatments at 19 °C for 7 days. Blue light and red light treatments were applied through using an LED blue lighting unit (430–470 nm) and LED red lighting unit (610–640 nm), respectively. The distance between LED lamps and the culture was 20 cm, and multiple lamps were applied (the distance between LED lamps is 10 cm). The light intensity was about 50 µmol/m^2^/s. After 7 days of the treatments, the stipes and pilei of mushroom in bottles were collected for RNA sequencing and metabolic measurements. RNA sequencing experiment and metabolome experiment have 3 biological replicates and 4–5 biological replicates, respectively. Each biological replicate is pool of all mushrooms from a pot. Measurement of fruiting body yield has three biological replicates (pots).

### Metabolome analysis

After 7 days of the light treatments, the 60 mg of mushroom sample was treated with a mix of 480 µL extraction solution (methanol:H_2_O = 3:1) and 20 µL internal standard solution (L-2-Chlorophenylalanine in 1 mg mL^− 1^). A mix of extraction solutions from each sample was used as quality control sample. The extraction solutions were dried using a vacuum concentrator without heating. The dried samples were incubated in methoxy amination hydrochloride (20 mg mL^− 1^ in pyridine) for 30 min at 80 °C, and then 70 µL of the BSTFA regent (1% TMCS, v/v) were added to the sample aliquots with incubating for 1.5 h at 70℃. All samples were loaded into a GC-TOF-MS system. GC-TOF-MS analysis was performed using an Agilent 7890 gas chromatograph system coupled with a Pegasus HT time-of-flight mass spectrometer. The system used a DB-5MS capillary column coated with 5% diphenyl cross-linked with 95% dimethylpolysiloxane (30 m × 250 μm, 0.25 μm film thickness). The mass spectrometry data were generated using full-scan mode with the m/z range of 50–500. Chroma TOF 4.3X software of LECO Corporation and LECO-Fiehn Rtx5 database were used to MS data analysis. *T* test (*P* < 0.05) was used to discover the differentially accumulated metabolites among dark, blue light, and red light treatments.

### RNA sequencing and qRT-PCR

After 7 days of blue light or red light treatment, the pilei and stipes of *F. filiformis* were exposed to RNA sequencing. The total RNA of mushroom samples was extracted with RNAprep Pure Kit (Tiangen, China). The RNA quality was evaluated using agarose gel and Agilent 5400 bioanalyzer (Agilent Technologies, USA). Sequencing libraries were generated using NGS Ultima Dual-mode RNA Library Prep Kit. After generating sequencing libraries, RNA sequencing was performed on Illumina planform (6G sequencing data). Genome sequence of *F. filiformis* was downloaded from NCBI with accession ASM1180015v1. Funannotate v1.8.15 software was applied to annotate the genome of *F. filiformis* (Palmer and Stajich 2016), and then the gene function was predicted with eggNOG mapper online version (http://eggnog-mapper.embl.de/). The annotated genome was used as the reference genome for the gene expression analysis. The reference genome index was established by HISAT2 v2.2.1(Kim et al. 2019). FeatureCounts was used to quantify gene expression levels (Liao et al. 2014). Differentially expressed genes (DEGs) were detected by DESeq2 (adjusted *P* value ≤ 0.05 and |log2fold change|≥1). RNA samples of each treatment and tissue were exposed to qRT-PCR according to method of Wang et al. 2020. *Actin* gene was selected as internal control gene. The primer sequences for tested genes were listed in Additional file 1: Table S1. The relative expression of the target genes was calculated using the △△Ct method (Livak and Schmittgen 2001).

## Results

### Growth

**Fig. 1:**
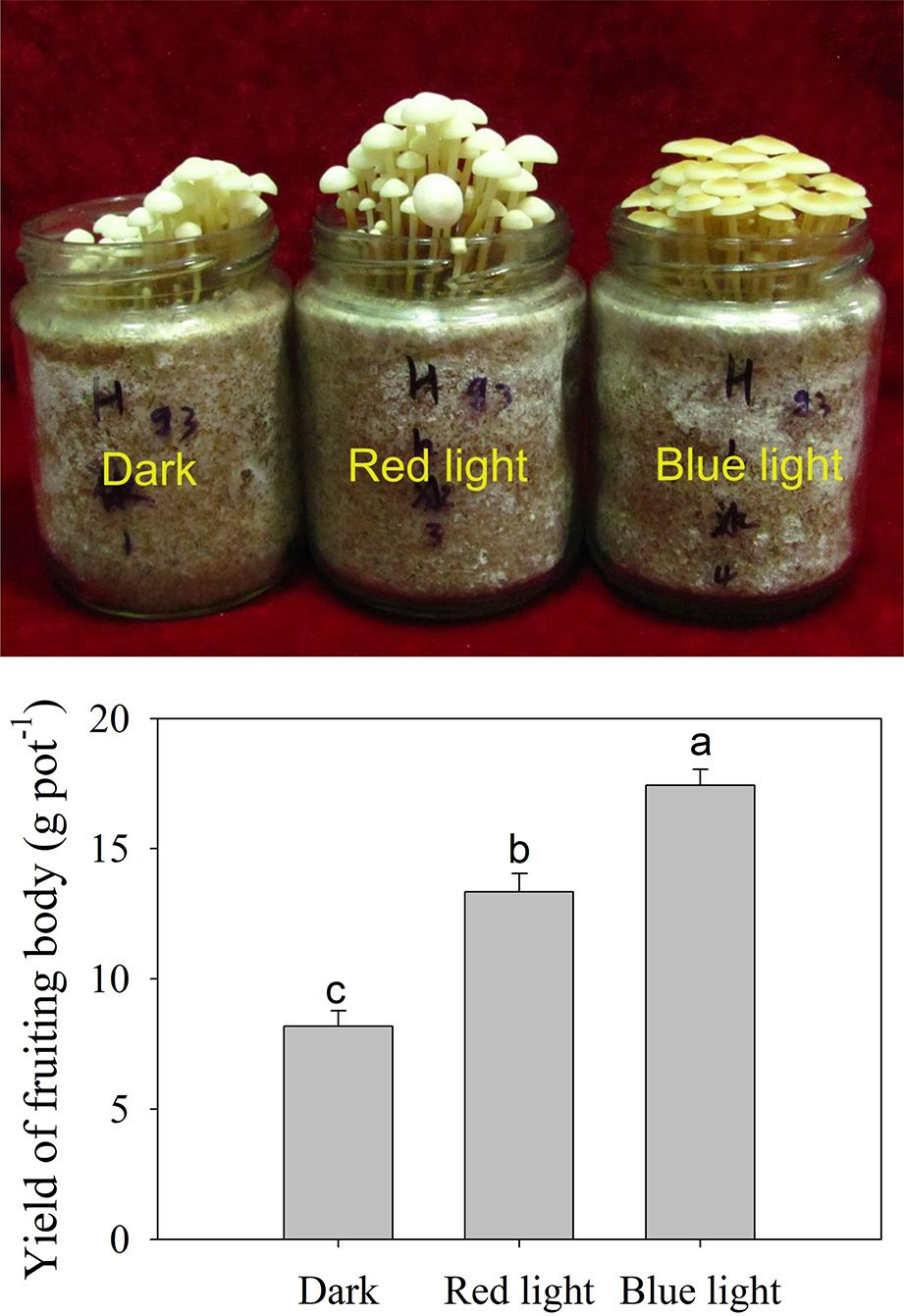
Growth status of *F. filiformis* under dark, blue light and red light conditions. Different letters above bar indicate significant differences among treatments (*t*-test, *P*-value < 0.05). Newly emerged primordium was exposed to blue light, red light, and dark treatments at 19 °C for 7 days. The values are means (± SD) of three biological replicates (pots)

The effects of dark, red light and blue light on morphogenesis of *F. filiformis* were recorded. The results showed that both red light and blue light increased the yield of fruiting body of *F. filiformis* compared with dark treatment, with greater enhancement of blue light treatment than red light treatment (Fig. 1). Blue light treatment also significantly induced pigmentation in the fruiting body.

### Metabolome analysis in pilei

**Table 1:** Effects of blue light and red light on concentrations of free fatty acids in the pilei of *F. filiformis*. Newly emerged primordium was exposed to blue light, red light, and dark treatments at 19 °C for 7 days

	Red light/dark			Blue light/dark			Blue light/red light	
	Fold change	*P* value		Fold change	*P* value		Fold change	*P* value
Stearic acid	1.006	0.965		0.747	0.255		0.742	0.306
Palmitic acid	0.934	0.678		0.872	0.549		0.934	0.773
Myristic acid	0.776	0.615		0.930	0.816		1.198	0.657
Linolenic acid	1.280	0.436		0.714	0.398		0.558	0.012
Linoleic acid methyl ester	0.958	0.766		1.191	0.583		1.243	0.487
Itaconic acid	1.119	0.852		3.524	0.065		3.148	0.083
Beta-Hydroxymyristic acid	1.038	0.787		0.757	0.033		0.729	0.028
Arachidonic acid	1.034	0.913		0.686	0.205		0.663	0.333
2-ketoadipate	0.765	0.424		0.963	0.923		1.259	0.609

We applied the metabolomics technology based on GC-TOF-MS to explore the metabolic response of *F. filiformis* to blue light and red light. Nine free fatty acids were detected in the pilei (Table 1). Red light treatment only produced slight effects on accumulation of free fatty acids in the pilei (Table 1), while blue light reduced the concentration of beta-hydroxymyristic acid in the pilei (Table 1). A total of 31 amino acids were detected in the pilei (Table 2). In the pilei, red light treatment reduced levels of L-allothreonine, alanine, histidine, and serine, while blue light treatment lowered the levels of saccharopine, 5-methoxytryptamine, and L-kynurenine (Table 2). Both light treatments enhanced the levels of lysine in the pilei (Table 2). In the pilei, blue light reduced concentration of digalacturonic acid and 4-hydroxybutyrate, but it enhanced accumulation in fumaric acid, D-glyceric acid, and 2-deoxytetronic acid (Table 3). Red light treatment inhibited the accumulation of 3-hydroxybutyric acid and 3-hydroxypropionic acid in the pilei (Table 3). In the pilei, we collectively detected the 47 carbohydrates under the three conditions. In the pilei, blue light treatment reduced the concentrations of fructose-6-phosphate, phytosphingosine, glucose-6-phosphate, glucose-1-phosphate, threonic acid, trehalose-6-phosphate, maltitol, pyruvic acid, 6-phosphogluconic acid, galactinol, alpha-D-glucosamine 1-phosphate, and fructose 2,6-biphosphate (Table 4). However, blue light treatment greatly enhanced levels of arbutin and sorbitol in the pilei, and red light treatment increased concentration of 1,5-anhydroglucitol and arbutin in the pilei (Table 4).

**Table 2:** Effects of blue light and red light on concentrations of amino acids in the pilei of *F. filiformis*. Newly emerged primordium was exposed to blue light, red light, and dark treatments at 19 °C for 7 days

	Red light/dark			Blue light/dark			Blue light/red light	
	Fold change	*P* value		Fold change	*P* value		Fold change	*P* value
Lysine	1,388,815	0.006		1,767,268	0.006		1.273	0.550
L-Allothreonine	0.688	0.011		1.013	0.941		1.474	0.120
Alanine	0.681	0.030		0.643	0.063		0.945	0.854
Histidine	0.749	0.041		0.869	0.275		1.161	0.406
Serine	0.821	0.049		0.931	0.565		1.134	0.420
Aspartic acid	0.531	0.053		0.899	0.831		1.694	0.419
Tyrosine	0.709	0.063		0.695	0.066		0.981	0.934
Glycine	0.597	0.077		0.939	0.791		1.573	0.133
Oxoproline	0.886	0.127		0.822	0.451		0.928	0.788
Aminomalonic acid	0.342	0.143		0.979	0.963		2.861	0.019
Valine	0.722	0.144		0.787	0.219		1.090	0.754
Beta-Alanine	0.509	0.155		0.670	0.376		1.318	0.601
L-cysteine	0.413	0.162		0.582	0.384		1.409	0.718
Glutamic acid	0.601	0.182		1.426	0.286		2.373	0.014
Citrulline	0.770	0.194		0.603	0.116		0.783	0.403
N-Carbamylglutamate	0.739	0.195		0.833	0.494		1.127	0.674
Asparagine	0.603	0.246		1.177	0.775		1.950	0.320
Phenylalanine	0.631	0.247		1.632	0.304		2.587	0.088
N-Methyl-DL-alanine	0.724	0.278		0.658	0.160		0.908	0.811
Norvaline	0.829	0.522		0.581	0.156		0.700	0.409
Tryptophan	0.800	0.550		0.895	0.743		1.118	0.695
3-Cyanoalanine	0.735	0.572		2.786	0.103		3.789	0.052
Isoleucine	0.838	0.586		1.286	0.388		1.534	0.032
Proline	0.862	0.623		1.159	0.595		1.344	0.065
Saccharopine	0.848	0.674		0.258	0.039		0.304	0.014
5-Methoxytryptamine	0.829	0.687		0.231	0.044		0.278	0.103
L-kynurenine	1.121	0.801		0.235	0.047		0.210	0.052
L-homoserine	1.104	0.848		1.187	0.619		1.076	0.839
3-Aminoisobutyric acid	0.940	0.868		0.751	0.406		0.798	0.633
Glutamine	0.878	0.929		2.775	0.172		3.160	0.118
Gly-pro	0.995	0.990		0.716	0.417		0.720	0.302

**Table 3:** Effects of blue light and red light on concentrations of organic acids in the pilei of *F. filiformis*. Newly emerged primordium was exposed to blue light, red light, and dark treatments at 19℃ for 7 days

	Red light/dark			Blue light/dark			Blue light/red light	
	Fold change	*P* value		Fold change	*P* value		Fold change	*P* value
3-hydroxybutyric acid	0.479	0.016		1.151	0.656		2.401	0.093
3-Hydroxypropionic acid	0.534	0.025		0.807	0.386		1.512	0.249
5-aminovaleric acid lactam	0.558	0.069		0.736	0.317		1.320	0.499
Glycolic acid	0.571	0.076		0.580	0.064		1.015	0.970
Glucoheptonic acid	1.150	0.077		0.984	0.819		0.856	0.004
Succinic acid	0.742	0.106		0.974	0.878		1.312	0.173
4-aminobutyric acid	0.723	0.152		0.776	0.179		1.073	0.779
Galactonic acid	1.550	0.158		1.330	0.338		0.858	0.410
4-hydroxybutyrate	0.538	0.160		0.372	0.035		0.690	0.490
Oxalic acid	0.709	0.198		0.882	0.626		1.245	0.554
Lactic acid	0.751	0.204		0.714	0.188		0.951	0.869
Succinate semialdehyde	0.648	0.267		0.669	0.277		1.032	0.944
Lauric acid	0.664	0.269		1.565	0.389		2.359	0.153
Adipic acid	0.706	0.325		0.284	0.104		0.403	0.193
3-hydroxyphenylacetic acid	0.170	0.327		3.088	0.246		18.128	0.085
Alpha-ketoisocaproic acid	0.606	0.339		0.367	0.063		0.606	0.440
3-Hexenedioic acid	0.709	0.364		0.246	0.078		0.347	0.124
Abietic acid	1.412	0.384		1.020	0.964		0.722	0.059
2-Deoxytetronic acid	1.932	0.389		6.308	0.005		3.264	0.022
Aconitic acid	0.730	0.433		2.066	0.101		2.832	0.068
Alpha-ketoglutaric acid	0.759	0.447		1.516	0.465		1.997	0.258
D-Glyceric acid	1.400	0.500		2.740	0.050		1.957	0.122
Digalacturonic acid	1.171	0.507		0.512	0.006		0.437	0.020
Maleic acid	0.822	0.685		1.693	0.224		2.060	0.079
Malonic acid	0.891	0.687		0.977	0.945		1.096	0.818
2-methylfumarate	1.316	0.697		3.296	0.058		2.504	0.116
Glutaric acid	1.281	0.732		2.335	0.108		1.823	0.241
Elaidic acid	0.856	0.760		0.632	0.309		0.738	0.597
Fumaric acid	1.029	0.812		1.499	0.021		1.457	0.041
D-galacturonic acid	0.926	0.852		0.711	0.331		0.768	0.460
5-Hydroxyindole-3-acetic acid	0.955	0.876		1.752	0.120		1.834	0.118

**Table 4:** Effects of blue light and red light on concentrations of carbohydrates in the pilei of *F. filiformis*. The differentially accumulated carbohydrates were displayed. Newly emerged primordium was exposed to blue light, red light, and dark treatments at 19℃ for 7 days

	Red light/dark			Blue light/dark			Blue light/red light	
	Fold change	*P* value		Fold change	*P* value		Fold change	*P* value
Ribulose-5-phosphate	1.802	0.098		0.260	0.168		0.144	0.001
Sorbitol	219	0.347		2121	0.000		9.697	0.002
Glucose-1-phosphate	1.096	0.621		0.434	0.003		0.396	0.002
Erythrose	0.796	0.669		1.855	0.057		2.329	0.006
Fructose-6-phosphate	0.897	0.601		0.343	0.001		0.382	0.009
Ribose-5-phosphate	1.173	0.586		0.719	0.367		0.613	0.013
Phytosphingosine	0.887	0.175		0.605	0.001		0.682	0.014
Glucose-6-phosphate	0.866	0.539		0.328	0.001		0.379	0.015
Lactulose	0.773	0.223		1.382	0.077		1.788	0.016
Gluconic acid	1.653	0.065		0.792	0.304		0.479	0.023
1,5-Anhydroglucitol	6.805	0.001		3.717	0.084		0.546	0.024
Threonic acid	0.670	0.203		0.126	0.004		0.188	0.024
Palatinose	2.625	0.160		0.309	0.342		0.118	0.030
Maltitol	0.908	0.623		0.526	0.005		0.579	0.031
Galactinol	1.384	0.246		0.631	0.017		0.456	0.041
trehalose-6-phosphate	1.017	0.936		0.556	0.005		0.546	0.058
Fructose 2,6-biphosphate	0.562	0.406		0.001	0.047		0.002	0.063
6-phosphogluconic acid	0.946	0.882		0.400	0.009		0.423	0.141
Alpha-D-glucosamine 1-Phosphate	0.942	0.776		0.595	0.035		0.631	0.167
Pyruvic acid	0.520	0.055		0.375	0.005		0.721	0.462
Arbutin	45,103	0.006		43,460	0.000		0.964	0.904

### Metabolome analysis in stipes

**Table 5:** Effects of blue light and red light on concentrations of free fatty acids in the stipes of *F. filiformis*. Newly emerged primordium was exposed to blue light, red light, and dark treatments at 19 °C for 7 days

	Red light/dark			Blue light/dark			Blue light/red light	
	Fold change	*P* value		Fold change	*P* value		Fold change	*P* value
Stearic acid	1.27	0.390		1.18	0.611		0.93	0.374
Palmitic acid	0.87	0.277		0.69	0.017		0.80	0.119
Myristic acid	1.20	0.574		0.95	0.897		0.79	0.054
linolenic acid	0.74	0.290		0.35	0.016		0.47	0.037
Linoleic acid methyl ester	1.00	0.992		1.26	0.502		1.26	0.167
beta-Hydroxymyristic acid	1.37	0.316		0.73	0.507		0.54	0.000
2-ketoadipate	1.54	0.514		1.32	0.628		0.85	0.764

**Table 6:** Effects of blue light and red light on concentrations of amino acids in the stipes of *F. filiformis*. Newly emerged primordium was exposed to blue light, red light, and dark treatments at 19℃ for 7 days

	Red light/dark			Blue light/dark			Blue light/red light	
	Fold change	*P* value		Fold change	P value		Fold change	*P* value
Lysine	575,010	0.193		1,560,172	0.000		2.713	0.055
Tyrosine	0.958	0.691		0.687	0.004		0.717	0.024
Tryptophan	1.183	0.529		0.549	0.006		0.464	0.047
Serine	0.971	0.843		0.703	0.060		0.724	0.030
Citrulline	0.749	0.116		0.720	0.078		0.962	0.845
Norvaline	1.987	0.517		3.615	0.132		1.820	0.317
L-cysteine	0.503	0.101		0.472	0.138		0.938	0.929
Asparagine	0.724	0.158		0.747	0.156		1.031	0.875
Histidine	1.010	0.956		1.185	0.163		1.173	0.413
Phenylalanine	0.859	0.491		0.686	0.171		0.798	0.083
Glycine	0.976	0.919		0.694	0.203		0.711	0.102
N-Methyl-DL-alanine	3.027	0.059		2.624	0.210		0.867	0.668
Glutamic acid	1.707	0.106		1.441	0.322		0.844	0.356
Glutamine	207,462	0.407		189,537	0.356		0.914	0.952
Proline	1.023	0.932		0.771	0.444		0.753	0.108
Isoleucine	0.906	0.734		0.769	0.451		0.849	0.208
Aminomalonic acid	1.501	0.294		0.697	0.500		0.464	0.031
3-Cyanoalanine	1.061	0.857		0.760	0.529		0.717	0.013
Glycine	1.797	0.315		1.407	0.541		0.783	0.496
L-homoserine	1.141	0.818		1.214	0.674		1.064	0.876
Alanine	1.380	0.502		1.283	0.678		0.930	0.719
Saccharopine	0.452	0.135		1.172	0.702		2.596	0.068
Aspartic acid	1.624	0.151		0.846	0.720		0.521	0.016
valine	1.573	0.256		1.148	0.790		0.730	0.292
L-kynurenine	1.167	0.473		1.077	0.808		0.923	0.741
5-Methoxytryptamine	1.621	0.395		1.099	0.841		0.678	0.452
Beta-Alanine	1.442	0.559		1.104	0.874		0.765	0.605
Gly-pro	1.380	0.291		0.943	0.882		0.684	0.061
3-Aminoisobutyric acid	1.727	0.276		1.106	0.884		0.640	0.245
Oxoproline	1.294	0.395		1.048	0.900		0.810	0.050
L-Allothreonine	1.294	0.397		0.975	0.949		0.753	0.022

Seven fatty acids were detected in the stipes. Red light treatment did not affect the accumulation of any fatty acids in the stipes, but blue light treatment reduced the contents of palmitic acid and linolenic acid in the stipes (Table 5). A total of 31 amino acids were detected in the stipes (Table 6). Light treatments had a great effect on the accumulation of amino acids in the stipes (Table 6). Blue light treatment resulted in reduction of the concentrations of tyrosine and tryptophan and a sharp increase in the levels of lysine in the stipes (Table 6). However, red light treatment did not affect accumulation of any amino acid in the stipes (Table 6). A total of 30 organic acids were detected in the stipes. The concentrations of glucoheptonic acid, aconitic acid, succinic acid, 5-hydroxyindole-3-acetic acid, and 3-hexenedioic acid were decreased by blue light treatment, and the levels of adienoic acid and 3-hexenedioic acid were decreased by red light treatment (Table 7). A total of 48 of carbohydrates were detected in the stipes. Red light treatment did not affect the accumulation of any carbohydrate in the stipes, whereas blue light treatment strongly affected the accumulation of many carbohydrates in the stipes (Table 8). Blue light treatment increased the concentrations of arbutin, 2-deoxy-D-glucose 2, 6-phosphogluconic acid, sorbitol, ribose-5-phosphate, and decreased the levels of d-glucoheptose, gluconic acid, and gentiobiose in the stipes (Table 8).

**Table 7:** Effects of blue light and red light on concentrations of organic acids in the stipes of *F. filiformis*. Newly emerged primordium was exposed to blue light, red light, and dark treatments at 19℃ for 7 days

	Red light/dark			Blue light/dark			Blue light/red light	
	Fold change	*P* value		Fold change	*P* value		Fold change	*P* value
Glucoheptonic acid	1.049	0.673		0.473	0.004		0.451	0.000
Digalacturonic acid	1.506	0.204		0.482	0.203		0.320	0.001
Aconitic acid	0.931	0.681		0.400	0.006		0.430	0.001
Alpha-ketoisocaproic acid	2.292	0.115		0.168	0.232		0.073	0.004
D-Glyceric acid	1.111	0.772		0.317	0.095		0.286	0.009
2-Methylfumarate	1.220	0.548		0.803	0.619		0.658	0.009
5-Hydroxyindole-3-acetic acid	0.970	0.897		0.476	0.038		0.491	0.017
Glutaric acid	1.056	0.878		0.643	0.384		0.608	0.043
Glycolic acid	1.571	0.260		0.908	0.873		0.578	0.057
Galactonic acid	0.211	0.150		0.742	0.618		3.514	0.068
Succinate semialdehyde	1.886	0.235		0.870	0.858		0.461	0.073
Adipic acid	0.572	0.030		0.924	0.685		1.614	0.079
5-aminovaleric acid lactam	1.988	0.283		0.461	0.317		0.232	0.081
Elaidic acid	1.402	0.542		0.415	0.372		0.296	0.087
Succinic acid	0.819	0.127		0.650	0.015		0.794	0.109
Lauric acid	1.093	0.679		0.811	0.504		0.742	0.219
Malonic acid	1.687	0.234		1.191	0.766		0.706	0.283
Alpha-ketoglutaric acid	1.546	0.662		0.335	0.302		0.216	0.303
2-Deoxytetronic acid	1.946	0.417		2.943	0.050		1.512	0.337
Lactic acid	1.469	0.262		1.136	0.790		0.773	0.343
Oxalic acid	1.471	0.340		1.168	0.782		0.793	0.356
3-Hydroxyphenylacetic acid	1.459	0.813		0.002	0.356		0.001	0.407
4-Hydroxybutyrate	2.081	0.309		1.325	0.665		0.637	0.486
3-Hexenedioic acid	0.599	0.020		0.653	0.039		1.091	0.595
3-Hydroxypropionic acid	2.452	0.152		2.044	0.432		0.834	0.704
3-Hydroxybutyric acid	1.428	0.502		1.156	0.855		0.810	0.706
maleic acid	0.735	0.503		0.659	0.387		0.896	0.709
Abietic acid	0.965	0.919		0.997	0.994		1.033	0.786
4-Aminobutyric acid	0.789	0.294		0.844	0.370		1.070	0.805
fumaric acid	1.479	0.443		1.439	0.478		0.973	0.908

**Table 8:** Effects of blue light and red light on concentrations of carbohydrates in the stipes of *F. filiformis*. The differentially accumulated carbohydrates were displayed. Newly emerged primordium was exposed to blue light, red light, and dark treatments at 19℃ for 7 days

	Red light/dark			Blue light/dark			Blue light/red light	
	Fold change	*P* value		Fold change	*P* value		Fold change	*P* value
d-Glucoheptose	1.042	0.710		0.512	0.005		0.491	0.000
Galactinol	1.872	0.052		0.516	0.265		0.276	0.000
Melibiose	1.788	0.112		0.637	0.400		0.356	0.004
Myo-inositol	0.991	0.908		0.783	0.079		0.791	0.005
Sorbitol	0.860	0.354		943	0.030		1096	0.014
Trehalose-6-phosphate	1.275	0.487		0.597	0.296		0.468	0.016
2-deoxy-D-glucose	575	0.407		2828	0.000		4.913	0.018
Conduritol b epoxide	1.275	0.158		0.573	0.079		0.450	0.021
Mannitol	0.397	0.361		1.707	0.238		4.297	0.025
Arbutin	6202	0.407		25,348	0.000		4.087	0.035
Maltitol	1.797	0.133		0.900	0.814		0.501	0.037
Gentiobiose	0.742	0.075		0.546	0.017		0.735	0.047
Ribose	0.875	0.749		0.417	0.214		0.477	0.049
Ribose-5-phosphate	1.280	0.709		2.463	0.050		1.925	0.058
Gluconic acid	0.889	0.719		0.301	0.006		0.339	0.059
6-phosphogluconic acid	1.684	0.362		2.565	0.017		1.523	0.239

### Gene expression

**Table 9:** Blue light upregulated genes in the pilei of *F. filiformis.* Key upregulated DEGs were displayed. Newly emerged primordium was exposed to blue light, red light, and dark treatments at 19℃ for 7 days

Gene ID	Log2Fold Change (Blue light/dark)	Gene function
FV_011140	1.7274	Oxalate decarboxylase
FV_011139	1.8992	Oxalate decarboxylase
FV_004194	2.1191	3-beta hydroxysteroid dehydrogenase/isomerase family
FV_014197	1.0177	Aryl-alcohol oxidase
FV_011530	1.2868	Carbohydrate esterase family 4 protein
FV_014457	1.2586	Enoyl-(Acyl carrier protein) reductase
FV_014231	1.2888	protein CC1G_08491 Coprinopsis cinerea okayama7 130
FV_002491	1.1768	cytochrome P450
FV_005242	1.0518	NAD(P)H-binding
FV_013107	1.0148	Enoyl-(Acyl carrier protein) reductase
FV_014126	1.6979	Ergot alkaloid biosynthetic protein a
FV_012322	1.4085	3-beta hydroxysteroid dehydrogenase/isomerase family
FV_010589	1.2283	Pectate lyase
FV_010765	1.4408	Synthase

We conducted transcriptomic analysis on the pilei and stipes of *F. filiformis* after 7 days of blue or red light treatment, and collectively detected 17,084 expressed genes. Seventy differentially expressed genes (DEGs) were discovered under blue light and dark treatments in the pilei, with 26 upregulated genes and 44 downregulated genes. Red light upregulated the expression of 30 genes and downregulated the expression of 204 genes in the pilei. In the pilei, blue light upregulated expression levels of many key metabolic genes, such as oxalate decarboxylase gene, 3-beta hydroxysteroid dehydrogenase/isomerase gene, aryl-alcohol oxidase gene, carbohydrate esterase gene, enoyl-(Acyl carrier protein) reductase gene, cytochrome P450 gene, and pectate lyase gene (Table 9). In the pilei, blue light also decreased expression levels of some important genes, such as *hsp90* gene, heat shock factor gene, dual specificity phosphatase gene, nucleotide exchange factor Fes1 gene, L-lysine 6-monooxygenase gene, and NADH flavin oxidoreductase gene (Additional file 2: Table S2).

We discovered 384 DEGs under blue light and dark conditions in the stipes, with 72 blue light-upregulated genes and 312 blue light-downregulated genes. In the stipes, red light upregulated expression of 14 genes and downregulated expression of 14 genes. Blue light upregulated expression of many important hydrolase genes, oxidase genes and polysaccharide synthetase genes in the stipes (Table 10). For example, blue light upregulated expression of the aryl-alcohol oxidase gene, copper radical oxidase gene, cytochrome P450 gene, squalene epoxidase gene, and multicopper oxidase gene in the stipes (Table 10). Blue light treatment also upregulated expression of 4 hydrolase genes in the stipes, including glycosyl hydrolase gene, cellulase gene, acid protease gene, and alpha/beta hydrolase gene (Table 10). The expression levels of A (1–6) glucan synthase gene and a serine/threonine phosphatase gene also were enhanced by blue light treatment in the stipes (Table 10).

**Fig. 2:**
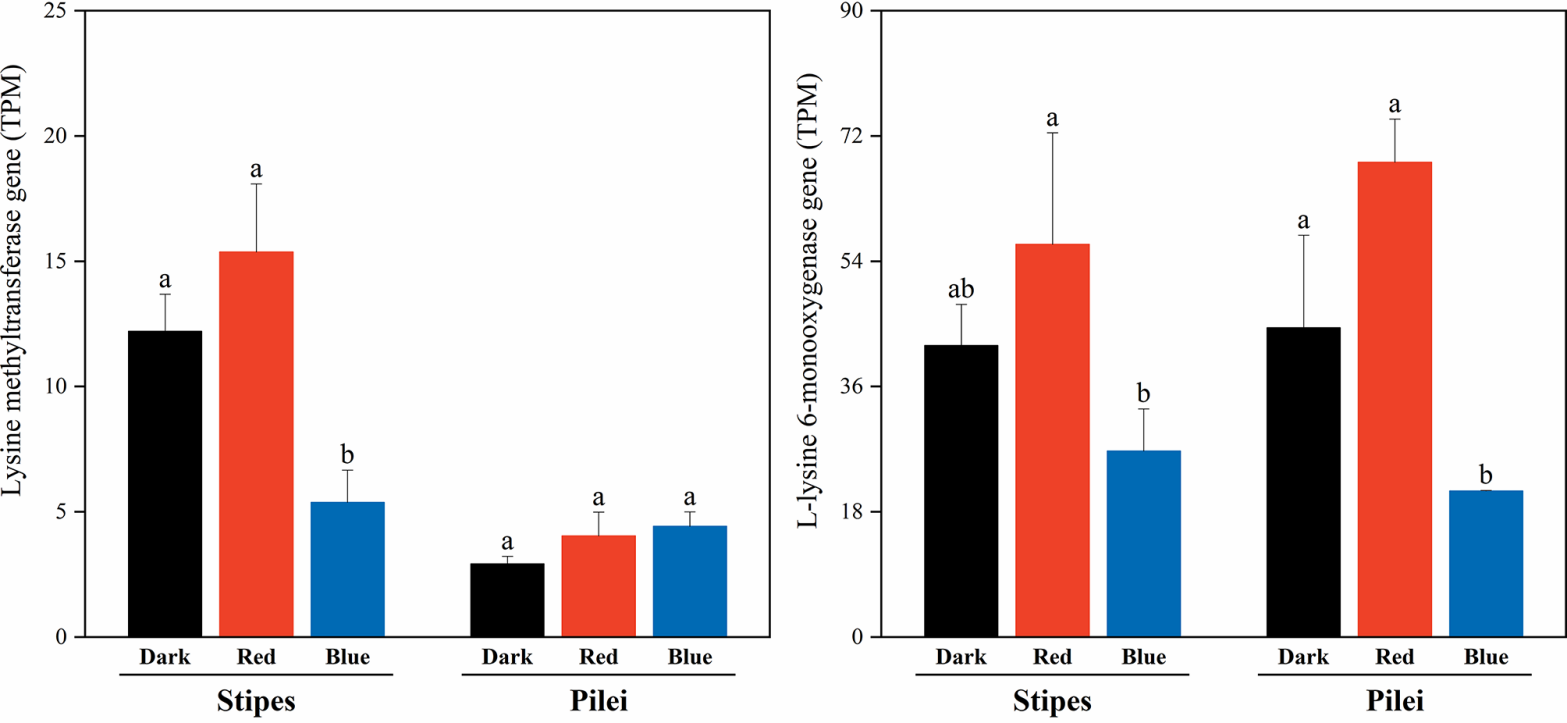
Effects of blue light treatment on expression of the genes involved in lysine degradation in *F. filiformis*. Different letters above bar indicate significant differences among treatments according to DESeq2 (adjusted *P* value ≤ 0.05 and |log2fold change|≥1). Newly emerged primordium was exposed to blue light, red light, and dark treatments at 19 °C for 7 days. The values are means (± SD) of three biological replicates

We focused on the expression response of the genes involved in lysine metabolism. Compared with dark and red light treatments, blue light treatment downregulated expression of lysine methyltransferase gene in the stipes but not in the pilei; however, blue light treatment downregulated expression of L-lysine 6-monooxygenase gene in both pilei and the stipes (no statistical significance between blue light and dark in the stipes)(Additional file 2: Table S2, Additional file 3: Table S3 and Fig. 2). We applied qRT-PCR to validate the results of the RNA sequencing analysis (Additional file 1: Table S1). In 9 of the 12 randomly selected genes, the fold changes of RNAseq analysis were similar to those from qRT-PCR experiment, indicating a reliable RNAseq experiment (Additional file 1: Table S1).

**Table 10:** Blue light upregulated genes in the stipes of *F. filiformis.* Key upregulated DEGs were displayed. Newly emerged primordium was exposed to blue light, red light, and dark treatments at 19℃ for 7 days

Gene Id	Log2Fold Change (Blue light/dark)	Function
FV_001217	1.078	3-keto sterol reductase
FV_003069	1.231	Acid protease
FV_016582	1.008	Aldo keto reductase
FV_001235	1.358	Alpha/beta hydrolase family
FV_014976	1.192	aryl-alcohol dehydrogenase
FV_014197	1.813	Aryl-alcohol oxidase
FV_001741	1.327	B-(1–6) glucan synthase
FV_003219	1.192	ABC transporter superfamily
FV_007632	1.043	MIP aquaporin (TC 1.A.8) family
FV_004196	1.218	Multicopper oxidase family
FV_009238	1.003	Peptidase M28 family
FV_012398	1.740	sterol desaturase family
FV_008928	1.069	Cellulase (glycosyl hydrolase family 5)
FV_000489	1.063	copper radical oxidase
FV_011377	1.503	Cytochrome P450
FV_002741	1.324	Cytochrome p450
FV_008223	1.559	ERG2 and sigma1 receptor-like protein
FV_014692	1.087	Ferritin-like domain
FV_006654	1.240	Glutathione S-transferase
FV_015074	1.097	Glycosyl hydrolases family 25
FV_011159	1.145	GMC oxidoreductase
FV_014812	1.112	high mobility group
FV_014190	1.157	Hydrophobins
FV_014189	1.061	Hydrophobins
FV_006653	1.602	Membrane bound O-acyl transferase family
FV_005026	1.008	MFS general substrate transporter
FV_010536	1.068	Microfibril-associated/Pre-mRNA processing
FV_004439	1.194	oxidoreductase
FV_001890	1.204	Serine/threonine phosphatases
FV_001500	1.367	Squalene epoxidase
FV_008428	1.152	Squalene epoxidase
FV_015210	1.037	Terpenoid synthase
FV_001916	1.670	to MEROPS metallopeptidase family M35
FV_001236	1.221	UbiA prenyltransferase family

## Discussion

Light is one of the most important environmental factors affecting the growth and development of almost all organisms. Previous studies have shown that appropriate light treatments can promote the fruiting body production of mushrooms (Kim et al. 2014; Yang et al. 2017; Wang et al. 2020) and change the shape of fruiting body of mushrooms (Park and Jang 2020), however, the mechanism underlying blue light response of mushroom remains incompletely understood. The present study also displayed that blue light treatment promoted growth and pigmentation of fruiting bodies of *F. filiformis*. The analysis of metabolic components indicated that fruiting bodies of *F. filiformis* contained a lot of amino acids, low molecular weight carbohydrates, flavonoids, terpenes, fatty acids, sterols and nucleosides, accumulation of which was strongly affected by blue light treatment. The present study explored the mechanism by which blue light regulates morphogenesis and nutrient accumulation of this mushroom. Blue light reduced the concentration of many low molecular weight carbohydrates in the pilei of *F. filiformis*, but it promoted the accumulation of a lot of low molecular weight carbohydrates in the stipes. Red light treatment produced a little effect on the accumulation of carbohydrates in the pilei and stipes of *F. filiformis*. Blue light treatment promoted the accumulation of many organic acids in the pilei of *F. filiformis*, but it decreased the accumulation of organic acids in the stipes of *F. filiformis*. For amino acids, blue light treatment inhibited the accumulation of tyrosine and tryptophan in the stipes of *F. filiformis*, but it promoted the accumulation of lysine in this organ. Red light treatment did not affect the accumulation of any amino acids in *F. filiformis*. The above data showed that the responses of pileus and stipe to blue light were obviously different. Lysine is an essential and important amino acid for human body. Lysine has nutritional value in promoting growth and development of human, enhancing immunity, promoting fat oxidation, and relieving anxiety. Our study revealed that blue light treatment can lead to the accumulation of a large amount of lysine in fruiting body of mushroom and increase its medicinal value, indicating important theoretical value for the industrial development of mushroom. The analysis of metabolome data displayed that blue light may shift the nitrogen source to lysine synthesis by inhibiting the accumulation of aromatic amino acids (tyrosine and tryptophan). Blue light treatment promotes the accumulation of beneficial metabolites by adjusting the direction of metabolic flow in *F. filiformis*. In addition, the decreased accumulation of organic acids in the stipes under blue light treatment may also shift metabolic substances and energy to the synthesis of carbohydrates and lysine in *F. filiformis*.

We applied RNA sequencing to explore the gene expression regulation mechanism underlying blue light response of *F. filiformis*. The results displayed that blue light treatment enhanced accumulation of lysine in fruiting body of *F. filiformis* through downregulation of two lysine degradation genes, lysine methyltransferase gene and L-lysine 6-monooxygenase gene (Fig. 2). Additionally, blue light upregulated expression of many important hydrolase genes, oxidase genes and polysaccharide synthetase genes in the stipes (Table 10). The upregulated expression of glycosyl hydrolase gene, cellulase gene, and acid protease gene may improve the ability of the stipe to biodegrade the medium, which elevates the growth rate of the fruiting body. In the stipes of *F. filiformis*, upregulated glucan synthase gene can promote the accumulation of polysaccharide and enhance its anticancer activity. Our result is different from finding in oyster mushrooms in which blue light promotes fruiting body production by enhancing respiration (Wang et al. 2020). Previous studies also showed that blue light upregulated the expression of cellulase gene in *Pleurotus eryngii* (Du et al. 2020) and the expression ofβ-glucosidase gene in *Lentinula edodes* (Kim et al. 2021). This suggests that different mushrooms respond to blue light with various metabolic regulation mechanisms.

In general, both blue light and red light can promote the fruiting body growth of *F. filiformis*. The response of pileus and stipe to blue light was different. In the stipes, blue light promoted the accumulation of low molecular weight carbohydrates and upregulated the expression of oxidase gene, hydrolase gene and glucan synthase gene, which improved the ability of the stipe to biodegrade the medium and elevated the growth rate of fruiting body. In the stipes of *F. filiformis*, blue light may shift metabolite and energy flow to synthesis of lysine and low molecular weight carbohydrates through inhibiting the accumulation of aromatic amino acids and organic acids, thereby enhancing its nutritional and medicinal value. This study revealed the metabolic and gene expression regulation mechanisms underlying blue light response of *F. filiformis*, which should promote the application of blue light in *F. filiformis* industry.

### Electronic supplementary material

Below is the link to the electronic supplementary material.

Supplementary Material 1

Supplementary Material 2

Supplementary Material 3

## Data Availability

All raw data of RNA sequencing are deposited at NCBI (Accession number PRJNA1012119). The mushroom materials and datasets used and/or analyzed during the current study are available from the corresponding author upon request.

## Abbreviations

DEG: differentially expressed genes
